# Intelligence

**Published:** 1831-12

**Authors:** 


### INTELLIGENCE.

### CHOLERA.

The following circular has been forwarded to the different Boards of Health in the metropolis.

*Council Office, Whitehall; 14th Nov.* 1831.

Sir: The Central Board of Health, having maturely weighed all the information which has been transmitted to them relative to the progress of the Asiatic spasmodic cholera in various parts of Europe, but more particularly guided by the conclusions on this head to which Drs. Russel and Barry have arrived after a five months' careful and laborious observation of the character of that disease in those parts of Russia which they have visited, beg leave to suggest for your consideration the following sanitary hints.

1. *As to Precautionary Measures.*

In order to ensure the adoption and realise the benefit of any system of sanitary arrangements in a large community, the first essential point is to divide that community into subordinate sections, and to form District Boards of Health, each to consist, if possible, of a resident clergyman and a number of substantial householders, and of one medical man at least.

These Boards should be charged with the following duties in their respective districts, viz.

1st. To appoint inspectors. Each inspector to visit daily, and to inquire carefully after the health, means of subsistence, cleanliness, and comforts of the inmates of, say, 100 houses more or less, according to local circumstances.

2dly. To receive and examine the reports of these inspectors, which should be made up to a given hour on each day.

3dly. To endeavour to remedy, by every means which individual and public charitable exertion can supply, such deficiency as may be found to exist in their respective districts in the following primary elements of public health, viz. the food of the poor, clothing, bedding, ventilation, space, cleanliness, outlets for domestic filth, habits of temperance, prevention of panic.

4thly. To report to their principal Boards respectively on the above heads, as well as on the actual state of health of their districts.

The subordinate divisions of each district ought to be numbered or lettered, and each district named; the names of the members of each Board, of the medical men attached to each, and of the visiting inspectors employed, should be placarded in conspicuous places.

Principal Boards of cities, towns, or parishes, to report directly to the Central Board in London: 1st, on the actual state of health of their whole population; 2dly, on the precautionary measures already carried into effect; 3dly, on the measures contemplated; 4thly, on suspected sources, if any there be, from whence this particular disease might possibly spring.

With regard to precautions as to intercourse with suspected or really infected persons or places, the Board are confident that good sense and good feeling will not only point out, but morally establish, as far as may be practicable, the necessity of avoiding such communication as may endanger the lives of thousands. But they strongly deprecate all measures of coercion for this purpose, which, when tried upon the continent, invariably have been productive of evil. The best inducements to a prompt acknowledgment of the disease having entered a family, as well as to an early and voluntary separation of the sick from the healthy, will always be found in the readiness and efficiency with which public charitable institutions attend to the objects noticed in s. 3.

It is with much satisfaction that the Board feel themselves authorised to declare, and it will no doubt be highly consolatory to the public to learn that, under proper observances of cleanliness and ventilation, this disease seldom spreads in families, and rarely passes to those about the sick, under such favorable circumstances, unless they happen to be particularly predisposed.

It will not therefore be necessary, where there is space, and where due attention is paid to cleanliness and purity of air, to separate members of families actually affected by the disease, nor to insulate individual houses, unless in cases of crowded, filthy, badly ventilated habitations, and other contingencies, which involve the health and safety of all.

It having been proved by ample experience, in more than one city in Europe, that the fitting-up and furnishing of hospitals for the reception of the poorer classes supposed likely to be attacked by the disease, at a period too long before its actual breaking out, has been productive of great waste of means, by the spoiling of various articles, and the consequent want of wholesome accommodation when most required, the Central Board would recommend that proper and sufficient houseroom only be secured and prepared in the first instance, and that the charitable be called upon only to *pledge* themselves to furnish, at a given notice, such articles of bedding, furniture, &c., or the value of them, as they would have at once contributed. By this means, the deterioration of perishable articles will be avoided, and, should the district entirely escape, the contribution will be saved.

The situation which the Board would recommend for temporary cholera hospitals, would be those most detached, insulated, and thoroughly exposed to free and open air; the description of house, such as would admit of the most perfect ventilation and cleanliness, and the largest space around the sick.

The Board would recommend, when a family is reported to be in an unhealthy state by the sub-inspector, and the disease confirmed to be cholera by a medical member of the District Board, that the head of such family, if unable to afford proper accommodation at home, be advised to send the sick person forthwith to the temporary hospital; and that the other members of the family be supplied with such additional means and comforts as their state may require, to enable them to resist the influence of the infected atmosphere in which they live.

2. *Medical and Dietetic Precautions.*

These will be found of considerable importance, from their contributing to prevent or diminish the susceptibility to infection which individuals may possess at the moment the disease breaks out.

No sudden nor extensive alterations should be made in the usual modes of living. All changes of food to be useful, indeed, not to be absolutely prejudicial, should tend to render it drier, more nutritive, and concentrated; moderately costive bowels, the almost invariable consequences of a dry, invigorating diet, will be found more conducive to exemption from cholera than an opposite habit.

Whenever aperients may become indipensable, those of a warm aromatic kind, in moderate doses, or domestic means, should alone be resorted to.

What is generally understood by salts, viz. Glauber's salts and Epsom salts, as well as other cold purgatives, should not be taken *in any quantities,* nor on any account without the express prescription of a medical man.

The medical members of the Board beg to state, in the most decided manner, that no specific preventive against cholera is known to exist; and that the drugs hitherto offered with this pretension, in countries where the greatest ravages have been caused by this disease, not only did not possess the negative virtue of doing no harm, but were found to be absolutely injurious.

The true preventives are a healthy body, and a cheerful, unruffled mind. Looseness of bowels should be immediately checked; and any thing like periodical chills or cold perspirations should be met by quinine, in suitable doses; but habitual drugging, at all times improper, is to be deprecated in the strongest terms when epidemic disease is apprehended.

The Board have been anxious to lay before the public, as early as possible, the above precautionary outlines, which they trust will tend, together with the suggestions emanating from the wisdom and observation of your and other local Boards, if not to exempt the whole population of these realms from the scourge of spasmodic cholera, at least to enable them to meet it, in the event of its appearing amongst them, with physical and moral constitutions the least likely to suffer from its virulence.

The Central Board will avail themselves of the earliest opportunity to transmit to you any further sanitary suggestions which may occur to them on the subject of precautionary measures, as well as an outline of instructions now in preparation for communities supposed to be actually attacked.

I have the honour to be, Sir, your most obedient servant,

E. STEWART, *Chairman.*

---

Drs. Hope and Sims have been elected Physicians to the St. Marylebone Parochial Infirmary; Dr. Hooper, who for many years filled the office with distinguished ability, having resigned.

---

Mr. Le Baume's *Portable Sudatory.* The ingenuity and simplicity of this apparatus deserve our favorable notice and the attention of the profession: by means of this "Sudatory," which is so light that it may be carried in one hand, the patient may be enveloped in air heated to any temperature that may be required, while a space is left open on one side, without exposing the body, in case friction should be deemed necessary. It may be used as a general or local hot air or vapour bath, and it possesses the great advantage that it may be used without moving the patient. The great benefit, and the frequently great difficulty, of inducing a free and genial perspiration by ordinary medical means are well known: Mr. Le Baume's "Portable Sudatory" is admirably adapted to produce this effect as well as many other equally to be desired in various severe diseases that will immediately suggest themselves to the mind of the medical practitioner.

APOTHECARIES' HALL, LONDON.

*September* 1831.

The Court of Examiners of the Society of Apothecaries of London, in conformity with their declaration of last year, have not made any alterations in their rules and regulations then published, although there are one or two points to which their attention has been more particularly directed, and which the lapse of another year may, perhaps, render it incumbent upon them to notice.

The Court thought it advisable last year to address some prefatory remarks to those most interested in the scheme of education which they were establishing, and they are induced, from various considerations, to take this occasion of extending their observations, and of again calling the especial attention of professional men, and also of the parents of youth destined to the profession, to several very important points that were but briefly noticed at that time. These observations are not founded upon theoretical views, but are the results of the extensive experience which the Court, acting under the directions of the Act of Parliament "for better Regulating the Practice of Apothecaries," have had afforded to them.

That the apothecary should have a sound and liberal education, and be practically acquainted with the duties of his profession, must be immediately apparent when the important and responsible nature of those duties is duly considered. The apothecary ministers to the great mass of the people in the function of the physician, and, by the usage of society, has the immediate superintendence of the public health entrusted to him: in the metropolis and the large provincial towns, he can be aided, in cases of difficulty, by the learning of the physician; but elsewhere he cannot avail himself of such valuable and desirable assistance, and in cases of danger, which are of frequent occurrence, he is obliged to rely exclusively upon his own resources, and can alone be sustained under his awful responsibility by a well-founded reliance on a knowledge of his profession.

The medical education of apothecaries was heretofore conducted in the most desultory and unsatisfactory manner: no systematic course of study was enjoined by authority, or established by usage; some subjects were attended to superficially and unprofitably, and others, of great importance, were neglected altogether. In their endeavours to remedy these defects, the Court of Examiners have been solicitous to proceed with the utmost circumspection, advancing progressively to the end they have in view, guided by their own experience, and aided by the suggestions offered by others for their consideration.

Before the student enters on his course of study more immediately professional, it is indispensably necessary that he should have received a classical education: in addition to the benefits resulting therefrom in the mental discipline it affords, a familiar knowledge of Greek and Latin is imperatively requisite to the medical student, since most of the terms of art employed in medicine and its associated sciences are derived from those languages, and his means of comprehending and retaining the information imparted by his teachers will materially depend on his acquaintance with them. That he may acquire the power of reasoning accurately on the complicated phenomena of life and disease, he will find the mathematical sciences of the greatest utility; and since many valuable contributions to professional literature have been made in the French and German languages, it is desirable, when opportunity offers, or circumstances will permit, that the youth should also be instructed in those languages, so as to be enabled to read and translate them with facility.

It is obvious that an education of this extent cannot be obtained in the limited time generally devoted to scholastic studies: the Court, therefore, advise that the apprenticeship required by the Act of Parliament should not be entered on till the age of seventeen, and that, during the two succeeding years, especial care should be taken to keep up and improve, by daily reading, the knowledge previously acquired.

Parents, in selecting a practitioner with whom to place their sons, should ascertain that he is legally qualified to practise as an apothecary, and also satisfy themselves that the nature of his engagements will permit him to regulate and superintend the studies of his pupil. A systematic course of study should be arranged, by which the pupil might be conducted progressively from elementary principles to the practical observance of disease; neither wasting his time by exclusive attention to pharmaceutic manipulations, nor employing it with as little profit in a premature attendance on the sick. He may thus be enabled, at the age of twenty-two or twenty-three, to present himself for examination. The law certainly allows him to undergo his examination at the age of twenty-one; but considering the nature, variety, and extent of his studies, and that he is not likely at that early period to have an opportunity of commencing practice, a little further delay will be no present sacrifice, and cannot fail to be productive of great eventual advantage to him.

In his attendance in the lecture room, and at the hospital, the student requires the friendly and experienced aid and guidance of his teachers; and it cannot be doubted that the learned individuals, who occupy the public chairs as lecturers, will be ready to afford their assistance, by pointing out to their classes the course of reading which will best illustrate their respective lectures; and also by ascertaining, in occasional examinations, the progress each pupil is making in his studies. The Court are aware that, in large classes, there are some attendant difficulties; but the advantages which would necessarily flow from a more intimate intercourse than has hitherto existed between lecturer and pupil would amply compensate for them.

In addition to these suggestions, there is another most important point to which the Court of Examiners solicit the especial attention of the physicians connected with hospitals and recognised dispensaries. Under their instruction, at the bedside of the patient, the student ought to learn the practical duties of his profession, by becoming acquainted with disease, not merely as it is described by authors, but as it actually appears in nature, under all the various modifications of sex, age, and temperament. The clear, distinct, and vivid impression made on the student's mind by clinical instruction, efficiently conducted, renders clinical teaching especially valuable, and the Court are most earnest in their desire that students should be encouraged to avail themselves of it with the utmost diligence.

The Court of Examiners have too much reason to know and lament, that, notwithstanding all their precautions, the attendance upon lectures, and more especially that upon hospital practice, is often grossly eluded or neglected; and they deem it their duty to express a hope that the teachers with whom the correction of this abuse must principally rest, will turn their attention to the removal of an evil of such magnitude. It would be competent for them to insist upon periodical signatures from their respective classes, proving that the pupils are actually in attendance; and it would be equally in their power entirely to withhold certificates from those who have neglected their attendance, or to qualify the testimonial in such a manner that the Court may apply to those who have been negligent of that degree of rigid scrutiny which the justice of the case might appear to demand.

Among the regulations of last year, the Court deemed it expedient to endeavour, by periodical registrations, to secure a consecutive and regular attendance on the several courses of lectures required by them. This important regulation the hall of the Society afforded them the ready means of carrying into effect with students at the schools in London; but without the assistance of gentlemen attached to those in the provincial towns, it could not have been adopted in them: the Court are anxious, therefore, to avail themselves of this opportunity of returning their thanks to those gentlemen at the several schools who so readily and cheerfully entered into their views, and who now give them their kind assistance by superintending the provincial registrations.

For information relative to the Regulations, medical students are referred to Mr. WATSON, who may be seen at his residence, 43, Berners street, between the hours of nine and ten o'clock every morning, (Sunday excepted;) and for information on all other subjects connected with the "Act for better Regulating the Practice of Apothecaries," application is to be made to Mr. EDMUND BACOT, clerk of the Society, who attends at the Hall every Tuesday and Thursday, from one to three o'clock.

It is expressly ordered by the Court of Examiners, that no gratuity be received by any officer of the Court.

---

*Prize proposed by the Phrenological Society of Paris:* To give an account of the positive knowledge *(les connaissances positives,)* which constitutes the science of Phrenology in its present state. Memoirs to be addressed to the Secrétaire général, rue de l'Université, No. 25, before the 1st June, 1832. Prize of the value of 500 francs.

---

### CONTAGIOUS NATURE OF CHOLERA.

IT was said by DIDEROT, that "incredulity is the first step to philosophy:" the anti-contagionists are certainly, then, in a fair way to become philosophers; for when facts, from the most undoubted authorities, are presented to them, they reply by asserting or insinuating that they are false. It is less difficult, however, to snatch a momentary appearance of victory in a medical society, by the aid of fluent diction, confident yet unfounded assertions, and a small sprinkling of wit, than it is to mislead the common sense of the profession, when they have time to reflect upon the evidence before them. It is quite impossible for us to collect *all* the evidence that might be adduced to shew that cholera is a contagious disease, but we submit sufficient facts to convince every unprejudiced mind:

"The sixth regiment of cavalry having left Ellore, where cholera did not exist, arrived at a place where it prevailed; and a squadron of the regiment having been necessitated, from the loss of their tents, to take possession of an old pagoda in the village, for shelter, cholera broke out in the corps at that place, and this squadron furnished almost every case of it."—*Hawkins on Cholera.*

"The prisoners in a jail have escaped cholera, while it prevailed all around them."—*Ibid.*

"Mr. Superintending Surgeon DUNCAN states, that when cholera appeared in the 34th regiment, on the route from Bellare to Bangalore, all the villages which they passed suffered from it immediately afterwards; and a native soldier, travelling from Bangadore to Nundidroog, at neither of which stations cholera had appeared, passed through the camp of the 34th regiment while the disease prevailed, was attacked by it, and died shortly after."—*Ibid.*

Dr. BURRELL says, in his Report, dated Seroor, July 27, 1828, "As every epidemic, by accumulation of subjects, has a tendency to propagate its virus, I am cautious in reporting this disease not infectious. *Almost every attendant in the hospital, in the short space of six days, has had the disease: there are about thirty attendants in the hospital.*"

"Many gardens and farms in the neighbourhood of Astrachan remained exempt from the epidemic, having broken off all intercourse with the diseased districts. In many villages, too, where similar precautions were taken, the issue was equally fortunate, although the cholera raged all around them."—*Hawkins.*

"Wherever measures were taken to prevent communication in the Russian dominions, there the disease has been totally checked, or has made but little progress. Petersburg has not escaped, because a strict quarantine has not been observed between it and Moscow."—*Ibid.*

"At Caramala-Gubeewa, some Russian peasants, living together, scarcely a hundred yards from the village, shut up their hamlet on the first report of the disease having appeared in the vicinity, and, by enforcing a strict quarantine during the prevalence of the epidemic, remained in health. The large establishment composing the academy of military cadets, at Moscow, was preserved by a similar plan from the scourge which was so active on all sides of it."—*Ibid.*

"M. De Lesseps, consul of France at Aleppo, when the cholera approached that city in 1822, retired, in company with all who wished to be of his party, to a garden at some distance from the city: his asylum was enclosed with walls, and was surrounded by a large fossé: there were only two doors, one for entrance, the other for going out. As long as the malady lasted, he admitted nothing from out of doors without submitting it to the precautions observed in lazarettoes. His colony consisted of 250 persons: *not a single individual contracted the disease;* while, at the very same time, within the city, 4000 persons perished in eighteen days."—*Kerandren sur le Choléra.*

"From every thing we have been able to learn as to the progress of cholera in the north of Europe; from its first appearance in the towns and villages of this country having been generally, if not always, preceded by the arrival of persons, or vessels, or both, from infected places; from the manner in which the disease has now broken out in this city (St. Petersburg), we see no other mode of accounting for its sudden appearance here, than by concluding that the barks from places on the Wolga, where the disease prevails, have brought something with them which, disseminated in this atmosphere, has been the immediate cause of the irruption of cholera which has just occurred."—*Drs. Russel and Barry.*

"There is at St. Petersburg a city prison, under the medical charge of Dr. Bish, an intelligent physician, and, previously to the appearance of the epidemic, an anti-contagionist. He, as well as all the other officers of the establishment, resides within the walls of the prison, by which all communication with the neighbourhood is prevented. On cholera appearing at St. Petersburg, all intercourse between the gaol and the town was rigidly cut off, and no instance of the malady occurred for some time; but at length the wife of one of the prisoners was admitted from one of the hospitals: she passed through the apartment where her husband was; she spoke to and saluted him, but proceeded in a moment to a part of the building appropriated to females. She had some diarrhœa when admitted, which on the following night proved to be cholera, and of which she died in twelve hours. Three women, who had been in the room with her, were next taken ill; and after them her husband, who also died. There were four hundred persons within the walls, and of these twenty-seven had cholera, which to fifteen proved fatal. *No case occurred in the portion of the prison allotted to nobles,* it being apart from the rest.—*Ibid.*

"It is a curious fact that another German colony, on the road to Moscow, between Yshna and Colpina, escaped, although the disease raged at both the places just mentioned; but it was observed that no travellers ever stopped at the German village, because the others afforded much better accommodation."—*Ib.*

"In a village of the government of Pensa, where this medical officer was sent in consequence of the breaking-out of the cholera, to trace its origin and afford medical aid, he learnt the following circumstances, which are attested by all the village authorities, and of which we are promised an authenticated copy, signed by himself. The son of a villager, who was coachman to a nobleman, at fifty versts' distance, died of cholera: the father went to the place, to collect the effects of the son, and brought home with him his clothes, which he put on, and wore a day or two after his arrival at his native village: he was shortly after seized with cholera, and died of it. Three women, who had watched him in sickness, and washed his body after death, were also seized, and died of the disease. The Doctor arrived in time to see the fourth case, and, finding that it spread on that side of the village, he had the common street barricadoed on the side where the disease had not reached, and interdicted all communication to the two sides of the village, even for the purpose of going to church. In that side in which the disease first broke out, upwards of one hundred cases of cholera occurred, of whom forty-five died; but the disease did not appear on the other side of the barricade."—*Dr. Russel.*

"While all around was infected, Sarepta, in which the quarantine regulations were most strict, escaped? and yet this disease is not called contagious!—*Quarterly Review, November.*

"In its visits to an uninfected country, it selects the principal port or frontier town, and from thence takes the most frequented thoroughfares to reach the largest cities. If the means of communication be rapid, the progress of the disease is rapid; if they be slow, the malady lingers in its march; if the distance be great, the time it takes to travel is proportionably so."—*Ibid.*

Moreau de Jonnés says, he has reckoned up, from the Madras, Bombay, and Bengal Reports, more than eighty instances of importation of cholera from one place to another, by corps of troops on their march.

It would be easy to adduce, from the various works before us, numerous facts in addition to those we have already given in proof of the contagious nature of cholera; but we deem it unnecessary to prolong our own labour, or to weary the patience of our readers, by an unnecessary accumulation of evidence. They who still maintain that the disease is not contagious might accuse us of injustice if we did not notice some of the arguments by which they endeavour to support their opinions: we shall chiefly refer to those we have recently heard advanced during the late discussions at the Westminster Medical Society.

It has been contended that cholera is not contagious, because so many persons escape who are exposed to contact with those labouring under the disease. The answer is obvious: Smallpox, measles, and scarlatina are admitted by all to be contagious, and yet how many individuals escape who live in the closest intercourse with patients affected with these maladies. Mr. Searle believes that cholera is not contagious, because certain persons have inoculated themselves with the blood of cholera patients, and tasted their evacuations, and have yet (marvellous truth!) escaped. Mr. Searle appeared serious while urging this very absurd argument; nor, indeed, can we suppose that he would sport with so grave a subject. These experiments, however, are themselves as ridiculous as their results are worthless; for diseases acknowledged to be contagious, as typhus, measles, &c., could not be transferred by any such means. Again, it is said that cholera may originate from some peculiar and modified action of heat, electricity, or climate: "but any such influence must be general, and therefore can never explain the facts which indicate the *partial* prevalence of the cholera, in a town to the exclusion of a suburb, in a suburb to the exclusion of a street, in a street to the exclusion of a house.*"

It is declared by the anti-contagionists, that most practitioners who have seen the disease assert that it is not contagious. This is one of the many instances we could mention of their lamentable inaccuracy, (to use the mildest term we can employ.) The fact is just the reverse, as is shewn by the Bombay, Madras, and Bengal Reports; and, independent of the evidence afforded by these documents, we may add the communication from Dr. Russel, that, "at a consultation of forty of the most respectable physicians of St. Petersburg, *thirty-eight* came to the conclusion, after mature deliberation on the documents laid before them, that the disease was infectious; and only two were of an opposite opinion." From Prussia, Austria, Sweden, and Holland, physicians were sent to the spot where cholera was raging, and they unanimously pronounced in favor of contagion.†

Another strong, or rather weak, hold of the anti-contagionists is that, if the disease were contagious, a greater proportion of medical men and hospital attendants than of other classes of society would be its victims. Upon this point we refer to the evidence of Drs. Barry and Russel: "At St. Petersburg, fifteen hospital physicians had been attacked, and six had died of cholera, up to the morning of the 13th July, out of 264 medical practitioners, of all descriptions, who were in the city at the breaking out of the present epidemic."—"Though we have not yet obtained official returns of the number, we are satisfied, from

* Quarterly Review, November.

† Med. Gazette.

the statements we have personally received in the numerous hospitals we have visited, that the proportionate number of attendants, of all descriptions, on the sick, who have been taken ill with cholera, is fully greater than that of the medical men."

We shall refer to only one more argument by which the anti-contagionists strive to maintain their position: they say that, during an irruption of cholera, individual cases frequently occur, without the possibility of tracing any communication between the sick and healthy. True: but how often are we unable to trace any inter-communication in cases of smallpox or plague; and yet who therefore denies the contagious nature of these diseases. Those who maintain that cholera is not contagious, call upon the contagionists for some *positive* proofs that it is transmissible from man to man: to this we reply, in the language of the very able writer in the Lancet, that "*multiplied* coincidences are tantamont to actual demonstration."

The following extract from Dr. Gooch's well-known paper, "Is the Plague a Contagious Disease?" also bears very forcibly upon this point:

"There are occasions when it is necessary to act on the supposition that a disease is contagious, though the evidence for this opinion fall far short of proof. The question is sometimes so difficult, life and health are so precious, and the precautions necessary to prevent the communication of the disease, if it should be contagious, comparatively such trifling evils, that a prudent physician will take care to be on the safe side, and act as if he were certain it was contagious, although, to an indifferent person, weighing the evidence in the nice scales of speculation, it would appear only a bare possibility: and here is the difference between a science, the subjects of which are inanimate things, like alkalies and acids, and a science, the subjects of which are flesh and blood, and health and life: that whereas in the former the *onus probandi* lies on him who affirms the proposition, because the disbelief of it leads to no injurious consequence; in the latter, the *onus probandi* lies on him who denies it, because the disbelief would occasion the neglect of measures which are harmless even if they be unnecessary, but the neglect of which may be fatal if they be essential."

---

### LITERARY INTELLIGENCE.

It is proposed to publish, by subscription, an English Translation of the Medical Works of Paulus Ægineta, the Greek Physician, illustrated with a copious Commentary, by Francis Adams, Surgeon. The work will be completed in three 8vo. volumes, handsomely printed in small type: it will come out in three distinct parts, at intervals of about four months, price 12s. the part. It is expected that the first part will come out the beginning of next year.

Two very elegant Engravings from the Portraits of Mr. Abernethy and Sir Astley Cooper, painted by Sir Thomas Lawrence, have recently been published: the likenesses are perfect.

### METEOROLOGICAL JOURNAL,

By Messrs. Harris and Co. Mathematical Instrument Makers, 50, High Holborn.

| Octob. | Moon. | Rain gauge. | Thermom 9 a.m. | max. | min. | Barometer. 9 a.m. | 10 p.m. | De Luc's Hygrometer. 9 a.m. | 10p.m | Winds. 9 a.m. | 10p.m. | Atmospheric Variation. 9 a.m. | 2 p.m. | 10 p.m. |
|---|---|---|---|---|---|---|---|---|---|---|---|---|---|---|
| 20 | | | 61 | 63 | 56 | 29.79 | 29.77 | 64 | 65 | SSW | SW | Cloudy | Fine | Fine |
| 21 | ○ | | 58 | 59 | 49 | .76 | .82 | 65 | 61 | WSW | WSW | Cloudy | — | — |
| 22 | | | 56 | 58 | 55 | .86 | .74 | 60 | 64 | SW | SW | Fine | Rain | Sho'ry |
| 23 | | | 60 | 60 | 49 | .78 | .85 | 66 | 64 | SW | SW | Cloudy | Rain | Fine |
| 24 | | .26 | 53 | 59 | 53 | .90 | .87 | 64 | 66 | SW | SSW | Cloudy | Fine | — |
| 25 | | | 58 | 61 | 55 | .68 | .46 | 66 | 64 | SSW | SW | Cloudy | Rain | Fine |
| 26 | | .06 | 59 | 59 | 53 | .34 | .43 | 63 | 64 | SSW | SSW | Cloudy | — | Cloudy |
| 27 | ☾ | .55 | 54 | 55 | 49 | .53 | .46 | 64 | 66 | SW | S | Rain | — | Rain |
| 28 | | .14 | 53 | 56 | 47 | .89 | 30.08 | 65 | 64 | SW | S | Fine | Fine | Fine |
| 29 | | | 51 | 55 | 46 | 30.10 | .09 | 63 | 63 | SW | WNW | Cloudy | — | — |
| 30 | | | 50 | 54 | 43 | .09 | .04 | 62 | 62 | | | Fine | — | — |
| 31 | | | 55 | 56 | 52 | .00 | .00 | 62 | 61 | WSW | SW | — | — | — |
| Nov. 1 | | | 55 | 56 | 53 | 29.83 | 29.71 | 61 | 62 | SW | SW | Cloudy | — | — |
| 2 | | | 54 | 55 | 43 | .57 | .51 | 64 | 63 | WSW | W | — | — | — |
| 3 | | | 50 | 50 | 38 | .30 | .35 | 61 | 61 | WSW | WNW | Fine | — | — |
| 4 | ● | | 45 | 51 | 45 | .57 | .69 | 61 | 62 | WNW | WSW | — | Cloudy | — |
| 5 | | | 50 | 50 | 47 | .32 | .35 | 64 | 64 | SW | WNW | Cloudy | Rain | — |
| 6 | | | 51 | 56 | 41 | .28 | .30 | 66 | 64 | SW | SW | — | Sleet | Sho'ry |
| 7 | | .11 | 48 | 50 | 45 | .30 | .37 | 63 | 61 | WSW | SW | Fine | Cloudy | Fine |
| 8 | | | 48 | 51 | 40 | .48 | .71 | 61 | 61 | W | W | — | — | — |
| 9 | | | 44 | 46 | 35 | .97 | 30.15 | 63 | 63 | WNW | NNE | Foggy | Fine | — |
| 10 | | | 39 | 53 | 38 | 30.22 | .10 | 63 | 64 | W | SW | — | Cloudy | Rain |
| 11 | | .20 | 53 | 55 | 50 | .05 | .10 | 67 | 66 | W | W | Rain | Fine | Fine |
| 12 | ☽ | | 52 | 55 | 49 | .15 | .12 | 66 | 65 | NW | WNW | Foggy | — | — |
| 13 | | | 50 | 51 | 34 | 29.80 | 29.80 | 60 | 59 | NW | NW | Sho'ry | — | — |
| 14 | | | 39 | 45 | 36 | .84 | .37 | 59 | 61 | W | W | Fine | — | Rain |
| 15 | | | 40 | 42 | 34 | .27 | .21 | 61 | 62 | W | W | Cloudy | Cloudy | Fine |
| 16 | | | 38 | 40 | 33 | .17 | .25 | 62 | 64 | WNW | WNW | Cloudy | Cloudy | Cloudy |
| 17 | | .35 | 35 | 35 | 30 | .41 | .39 | 63 | 65 | W | ESE | Foggy | T. Fog | F.& Sl. |
| 18 | | | 33 | 40 | 31 | .51 | .64 | 65 | 68 | NW | W | — | Cloudy | Fine |
| 19 | ☉ | | 40 | 41 | 35 | .28 | .42 | 67 | 65 | WNW | WNW | Cloudy | Cloudy | Cloudy |

The quantity of Rain fallen, in the month of October, was 3 inches and 69.100ths.

